# Innovative Digital Technologies for Purchasing and Consumption in Urban and Regional Agro-Food Systems: A Systematic Review

**DOI:** 10.3390/foods10020208

**Published:** 2021-01-20

**Authors:** Antonella Samoggia, Francesca Monticone, Aldo Bertazzoli

**Affiliations:** Department of Agricultural and Food Sciences and Technologies, University of Bologna, Viale Fanin, 50, 40127 Bologna, Italy; aldo.bertazzoli@unibo.it

**Keywords:** digital platforms, digitalisation, mobile phone applications, urban food systems, local food systems, systematic review

## Abstract

The use of digital technologies in the agro-food sector is growing worldwide, and applications in the urban and regional food systems represent a relevant segment of such growth. The present paper aims at reviewing the literature on which and how digital technologies support urban and regional agro-food purchasing and consumption, as well as their characteristics. Data collection was performed on Scopus and Web of Science. Articles were selected using a research string and according to specific inclusion and exclusion criteria. The Preferred Reporting Items for Systematic Reviews and Meta-Analyses (PRISMA) flow approach was adopted to explain data screening and selection. The 57 resulting studies were included in the final qualitative analysis, which explored the characteristics of the research studies and of the digital technologies analysed. Most of the studies analysed concerned the implications of digital technologies on local food consumption, especially focusing on consumption, primary production and hotel-restaurant-café-catering sector (HORECA), and to a limited extent on the retail sector. Consumers and farmers are the main targets of Information and Communications Technology (ICT) tools, whose principal aims are providing information on agro-food products and enhancing networking along the food supply chain. Analysing digital technologies allows a better understanding of their most popular features in order to support their spread among citizens. Digital technologies, and particularly Apps, can be a valuable instrument to strengthen agro-food chain actor relations and to promote urban and regional food systems.

## 1. Introduction

As reported by the Food and Agriculture Organisation (FAO), digital technologies can significantly contribute towards addressing the challenges faced by the global agro-food systems, at every level of the supply chain [1,2]. The FAO argued that digital technologies at farm level, such as sensors, robots and drones, can provide precise information to farmers and help them increase yields in a climate-friendly way. Blockchain technology can enhance traceability and sustainability by monitoring the food chain from the field to the final consumer [3]. The United Nations also explored the opportunities offered by digital technologies in the field of nutrition and concluded that they are helpful in providing tailored health advice but warned against their potential threats to the privacy of health information [4]. The FAO argued that “digital technologies can trigger major changes or “disruptions” in the food system that not only improve efficiency and speed, but also redistribute information and power along the value chain” [3]. A similar approach was adopted by the European Commission, which, albeit recognising the limited spread of digital technologies across the Union, considers them capable of increasing sustainability in the agro-food system [5] thus prioritising digitalisation in the Common Agricultural Policy (CAP) reform 2021–2027 [6].

The present review aimed at analysing the current literature on digital technologies for purchasing and consumption in urban and regional agro-food systems. In particular, the study aims at understanding:

1. How digital technologies reinforce the connection between urban and regional food systems.

Since more than 50% of the global population lives in cities, the FAO launched the City Region Food Systems (CRFS) Programme aimed at reinforcing rural-urban connections to strengthen regional agro-food systems. The literature agrees that CRFS initiatives foster sustainability in their local area. In fact, the benefits of CRFS initiatives range from increased food and nutrition security, to jobs and income creation, as well as climate change counteraction and better natural resources management [7,8,9]. Since 2010—and particularly after the 2015 Milan Food Policy Pact—hundreds of cities implemented food policies to support sustainable initiatives at local level [10]. Albeit such increasing interest, the role of digital technologies in strengthening local food systems is understudied and this research aims at filling this gap.

2. How digital technologies change food shopping experiences for consumers and food selling for producers and retailers.

E-commerce has seen a stable growth over the last few decades. In the European Union, the percentage of individuals who purchased goods on the internet at least once in one year increased substantially, rising from 36% in 2010 to 60% in 2019 [11]. The agro-food sector is still underdeveloped in this matter, but online grocery shopping grew in the last decade: in 2018, 15% of the European population purchased food on the internet at least once in the previous year, compared to 5% in 2009 [12]. The Covid-19 pandemic enhanced such habit, as 30% of consumers globally claimed to have started using e-commerce during lockdown [13]. Albeit such an increase in use, the research literature on digital technologies in the agro-food sector does not address all dimensions, being more focused on farm management than on shopping experience [2]. When focusing on the latter, the literature prioritises the analysis of consumer preferences and behaviours [14] to the characteristics of the available digital technologies. Therefore, this research aims at filling this gap and analysing the features of available digital technologies in the agro-food sector.

3. The characteristics of mobile phone Apps, and to highlight App differences compared to other digital technologies.

Among the available platforms for e-commerce, mobile phone Apps were chosen as a focus because of the following reasons. The predicted number of mobile phone users worldwide stands at 3.8 billion people in 2021 [15]. This data is matched by a consistent number of App downloads: in 2019, consumers downloaded 204 billion Apps to their devices [16]. Among all the available Apps, the Food and Drink category represents a share of 3.5%, while Health and Fitness 3.4% [17]. Healthy lifestyle Apps are the most studied, as they are considered a low-cost and efficient technology to promote behavioural change and improve people’s diets [18,19,20,21,22,23,24]. The use of Applications to strengthen the link between the consumer and other agro-food system actors was less studied [25], and the present research aims at filling this gap.

The article is structured as follows. Section 2 illustrates the methodology, describing data collection and data analysis. Section 3 presents the results of the literature review. Section 4 discusses the findings and limitations of the papers’ analysis, and suggests further areas of research.

## 2. Materials and Methods

### 2.1. Data Collection

**Figure 1:**
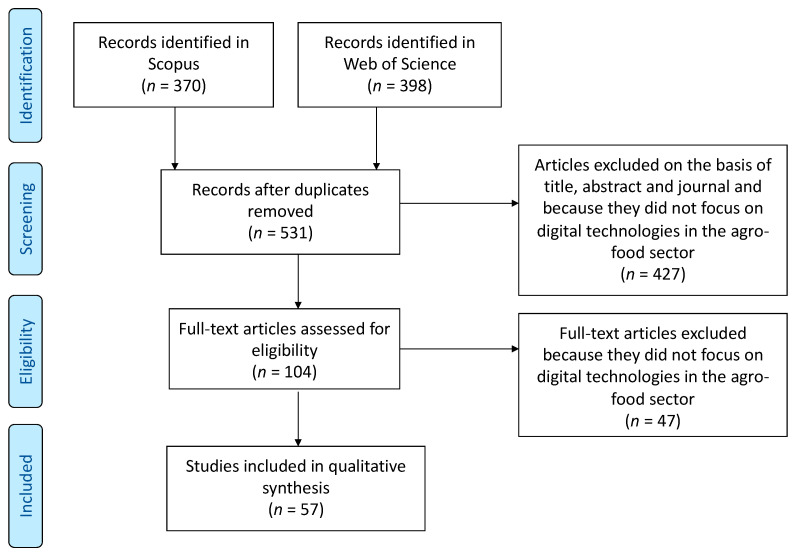
The Preferred Reporting Items for Systematic Reviews and Meta-Analyses (PRISMA) flow diagram outlines the literature review process.

The literature review of past studies on digital technologies in the agro-food sector was conducted in a structured and systematic manner. Figure 1 shows the search strategy process according to the four steps of the PRISMA (Preferred Reporting Items for Systematic Reviews and Meta-Analyses) flow diagram [26].

As shown in Figure 1, studies included in the literature review were retrieved from two scientific search engines: Scopus and Web of Science in April 2020.

A search string with a combination of selected search terms was used in both databases to yield relevant results (Table 1). The final string consisted of four groups of words.

The first group included digital application synonym words, such as “app”, “mobile”, “device”, “digital”, “smartphone Application” and “ICT”. ICT tools stand for Information and Communication Technology tools [27]. It is a broader term for Information Technology (IT), however, there is no universally accepted definition of ICTs considering that the concepts, methods and tools involved in ICTs are steadily evolving [28].

The second group of words focused on the agro-food sector using three words: “food”, “agro-food” and “agri-food”. Then the search included the terms “urban”, “local” and “regional” to identify urban and regional digital approaches. The fourth group of words aimed at limiting the selection to those digital applications that favour the exchange between producers and consumers: “consumer”, “consumption”, “marketing”, “commercialization”, “distribution”, “selling”, “delivery” and “trade”. Given the large number of papers from the field of medicine on the topic, AND NOT and “medic*” were added to exclude them as not relevant for the scope of the present research. As technology is a fast-changing field, a temporal limitation was put in place to include only research studies published between 2010 and 2020.

The search with the string on Scopus and Web of Science yielded 370 and 398 results, respectively. After the elimination of duplicates, 531 articles were screened based on title, abstract and journal of publication. The resulting 104 papers were read in full and 47 were eliminated as out of scope for this research, as they were not focused on agro-food products and on digital technologies. In total, 57 studies were therefore included in the final body of literature analysed.

### 2.2. Data Analysis

The selected articles were analysed by two researchers, in order to limit the possible bias. All the 57 research studies were analysed according to the following two main categories.

― Research studies characteristics:
  ― temporal and geographical distribution;
  ― fields of research;
  ― aim of the study;
  ― focus on urban and rural areas.
― Digital technologies characteristics:
  ― types of digital technologies;
  ― aims of digital technologies;
  ― section of the agro-food chain where digital technologies operate;
  ― key features of digital technologies.

## 3. Results

### 3.1. Temporal and Geographical Distribution of the Research Studies

**Figure 2:**
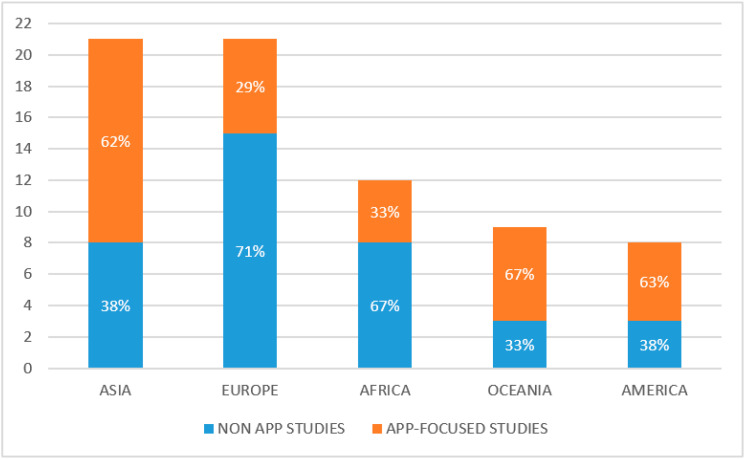
Geographical distribution of analysed studies (*n* and percentage of studies focused on Apps and not focused on Apps). Note: research studies that focused on more than one country were counted more than once.

Research works were well distributed around the world (Figure 2), with two continents standing out for the highest number of studies on digital technologies in the agro-food sector: Europe and Asia, with 37% of articles each. In total, 21% of the studies are focused on African countries, while 16% referred to Oceania and 14% to America (Table 2). European, Asian and American studies focused primarily on the consumer side of the food supply chain, researching the effects of digital technologies on local and sustainable consumption (Europe) and on healthy eating (America). Asian studies equally focused on sustainable consumption, healthy eating and the use of technologies in the Hotel-Restaurant-Café-Catering sector (HORECA). African researches mainly addressed the use of ICT tools by farmers, especially for farm management and marketing of products. For example, the “CocoaLink” platform connects stakeholders in the Ghanaian cocoa supply chain, “Digital Green” shares good agricultural practices among smallholder farmers in Ethiopia through videos in local languages, and “MFarm” connects Kenyan farmers with urban markets [29].

When selecting only studies about Apps (25 papers out of 57, 44%), a slightly different geographical distribution emerged (Figure 2). Asia was dominating the literature, with 13 articles [29,36,51,53,54,55,56,57,59,62,63,65,66], while Europe focused [29,35,36,38,48,49] and Oceania focused [29,36,76,77,79,81] followed with six studies each.

**Figure 3:**
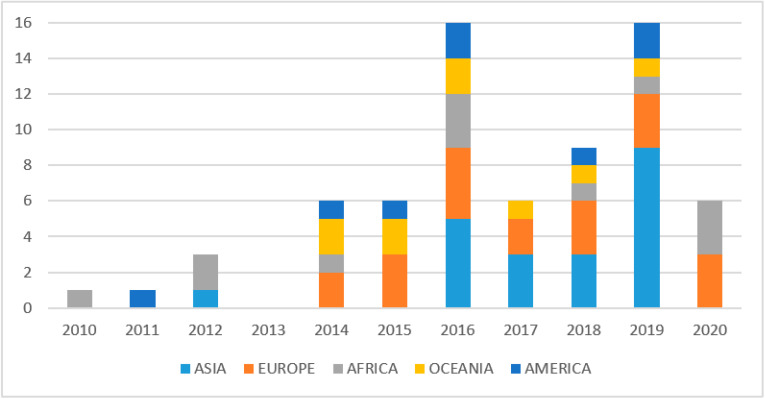
Historical evolution of published studies (*n* of studies).

The time frame 2010–2020 was effective in capturing a significant body of literature on the topic. From 2015 onwards, the body of research on digital technologies in the agro-food sector has grown consistently (Figure 3). It particularly peaked in 2019 when 16 papers on the topic have been published, a trend that may continue in the years ahead, considering that the database search for 2020 only included papers available until April.

Combining geographic and time distribution, results support that all five continents reflect the general time pattern mentioned above where after 2015 the body of research increased consistently (Figure 3). However, while for some continents (namely Europe and Asia) this meant a steady growth, for others (namely America and Oceania) it was only a small increase. Africa classified in the middle, with two peaks (in 2016 and in 2020) and a general increase in the remaining years. While print and broadcast media were the types of digital technologies on which research focused on the beginning of the decade (2010–2011), websites, social media and Apps made their appearance in 2012–2014 and grew steadily after that.

### 3.2. Research Studies′ Fields and Aims

The selected articles were well distributed in different disciplines, with IT being the most present (21% of research works) followed by the health and nutrition field (16%). The number of papers in the field of IT reflected the focus of the present research on digital technologies, while the high number of nutrition-related papers reflected a strong presence of nutrition ICT tools, particularly Apps. It was found that 10% of articles were in the field of agriculture. This finding may suggest limited research on IT in the primary sector, which is however increased in the past five years. In total, 3% of research works are published in a tourism-focused journal, which shows a new tendency of that field towards technology.

As summarised in Table 3, the three most frequent aims of the research studies were:

― the implications of digital technologies on local and sustainable consumption (35% of research studies);
― the effects of digital technologies on healthy eating (26% of research studies);
― the effects of digital technologies on farm marketing (19% of research studies).

**Table 3:** Aims of the research studies.

	Aims of the Studies	*n* of Studies	References
1	Study the implications of digital technologies on local and sustainable consumption	20 (35%)	[29,31,32,34,35,36,38,40,41,42,45,47,52,53,56,59,62,67,77,78,79]
2	Study the effects of digital technologies on healthy eating	15 (26%)	[30,37,38,43,55,58,61,72,74,76,80,81,83,84,85]
3	Study the effects of digital technologies on farm marketing	11 (19%)	[29,33,35,39,41,46,56,60,71,78,79]
4	Study the role of digital technologies in farm management	7 (12%)	[29,50,51,68,69,70,75]
5	Study the effects of digital technologies on the HORECA sector	5 (9%)	[55,63,64,66,73]
6	Present an App prototype	4 (7%)	[49,53,57,83]
7	Study the effects of digital technologies on tourism	3 (5%)	[42,44,48]
8	Study the role of digital in the retail sector	2 (4%)	[65,82]

Research studies that focused on more than one aim were counted more than once.

The vast majority of articles studying the implications of digital technologies on local and sustainable consumption were about online marketplaces, in the form of platforms and Apps. In most cases, these digital tools helped shorten the food supply chain, eliminating the middlemen and putting in direct contact primary producers and consumers. Limited attention is given to sustainability in the food service. In the relevant studies the digital technologies adopted were Apps or websites [53,59,77]. All articles studying the implications of digital technologies on local and sustainable consumption were recent (from 2014 onwards).

Articles on healthy eating were studying the efficacy of different digital tools (such as Apps, SMS, voice calls, websites) in nudging citizens toward more balanced diets. In most cases, this was achieved through a comparison of different kinds of interventions tested on a specific cluster of people.

Similarly to the studies on local and sustainable consumption, articles on the effects of digital technologies on farm marketing addressed the topic of e-commerce. However, this group focused on the farm-side analysing websites such as “Molisanissimo” and “AgroLink”. The former offers communication and promotion services to farms and food companies producing traditional products in an Italian region, by improving their positioning on the market [46]. The latter provides information to the farmers about the price of agricultural items sold at the agro-food markets in Bosnia and Herzegovina, so to better know their competitors [33].

The fourth most significant aim is the role of digital technologies in farm management, which is prevalent in African studies, as mentioned in 3.1. Articles in this category addressed not only how digital technologies can increase yields but mostly how they can enhance the creation of regional markets and the cooperation among farmers to strengthen their position in the food supply chain.

The analysis of the effects of digital technologies on the HORECA sector was the fifth most significant aim of the analysed studies. The vast majority of these articles focused on Asian countries, with specific interest on HORECA. In particular, the studies addressed if digital technologies help restaurants run their business [63], what is the consumers’ perceptions of food delivery services [66], and another study classified and evaluated catering establishments according to relevant categories [64].

The sixth most frequent research study category′s aim is the presentation of an App prototype [49,54,57,83]. The analysed prototypes were quite different from each other: Gilliand et al. [81] designed an App to encourage healthy eating by reducing educational, behavioural and economic barriers to accessing healthy and local food, while “FoodieRoute”, the App described by Fauzi et al. [54], guides tourists to discover eating places in the region of Sarawak in Malaysia. Gupta et al. [57] developed a mobile phone Application which allows farmers in India to sell their livestock more easily, while the FoodTrack study presented by Poelman et al. [49] allowed the researchers to examine the participants′ food environment to assess their food choices and to examine the moderating role of individual and contextual determinants (such as mood, companion, time of the day) of food purchases and consumption.

The three studies on the effects of digital technologies on tourism explored how social media, blogs and Augmented Reality Apps can attract tourists and improve their experiences [42,44,48]. All three solutions were proved key in local promotion strategies, as able to tell a story and involve tourists emotionally.

The least studied topic was the role of digital in retail, which was the focus of two studies only [65,82]. This result could be due to the difficulties in collecting information and data on retailers′ digital technologies content, as it may disclose information on retailers’ management strategies. Furthermore, retailers tend to process data and to conduct their research independently. One of the three case studies analysed by Syaglova [65] was a mobile Application of the Russian network “X5 Retail Group”, including 13,000 trading companies, operating as hypermarket and supermarket: such Application allowed buyers to use a loyalty card in electronic form, to receive special offers and recommendations on products and prices. The retail sector may need further studies to understand consumers’ perceptions of digital technologies use.

### 3.3. Focus on Urban or Rural Areas

In total, 44% of the selected articles focused on rural areas while 47% focused on urban settings. The latter reflects a general trend in the literature of exploring ICT tools in urban agro-food systems, supported by the United Nations interest on the topic [1,3,4]. Articles on cities were generally more focused on consumer behaviours and engagement, while the rural ones analysed digital technologies from the producers′ side. One study compared an urban and a rural experience: a London-based veg box scheme (Transition Belsize Veg Bag scheme) and an e-agriculture pilot project with Kenyan farmers [32]. Both cases support that the use of digital tools to document and share food-related projects allows the engagement of otherwise distant stakeholders and a stronger collaboration along the chain. This represents an innovative effect of technology adoption, which is usually relegated—especially in the farming sector—to the improvement of techniques [32].

### 3.4. Types of Digital Technologies Studied in the Research

Around half of the articles analysed Apps (44%). The other most frequently studied digital technologies were websites and platforms (35%), mobile phones and tablets (16%), social media (11%), print and broadcast media (4%)—such as newspapers and radio, videogames (2%) and modern kitchen appliances (2%) (Table 4).

One third of the Apps studied provided health advice, by helping with meal planning [76], tracking food purchases and consumption [49,55] and providing information on healthy food [81]. Almost another third of articles on Apps is focused on primary production, from yields improvement to stakeholders’ cooperation. “FarmDroid” a web Application studied by Henriques and Kock [69] enables smallholders to interact with traders, retailers, consumers, and each other; it also included a dashboard of local prices, planting and harvesting risk indices, weather conditions and access to a virtual marketplace.

The studies exploring websites and platforms focused on e-commerce, namely the buying and selling of food products using online platforms. Most of the studies investigated small-scale producers′ capability to adopt e-commerce selling strategies. One study was focusing on retailers to analyse whether costumer stickiness is different online and offline [67]. Half of the studies analysed online selling platforms in Europe, followed by Asia and Oceania. The two most studied online platforms were the Food Assembly and the Open Food Network: the former was the focus of two Italian researches on customers’ behaviour [45,47], while the latter was studied in two Australian studies [78,79] and one UK article [40] addressing producers′ benefits in joining the platform. E-commerce was not the only focus of online platforms: Bakırcı-Taylor et al. [84] compared the nutrition-advice website “Jump2Health” with other two types of nutritional interventions (phone text messages, Facebook page) to test with a randomised control trial which intervention resulted in a higher intake of fruits and vegetables in young children. Bassano et al. [44] compared two tourism-related websites “Umbria on the blog” and “Il Mangiastorie”: “Umbria on the blog” was considered more effective in attracting tourists as it reported the experiences of ten bloggers invited to visit the region for a weekend, while “Il Mangiastorie” simply told stories of traditional products [44]. Ahmed et al. [51] analysed “E-agriculture”, a platform where Bangladeshi producers can ask questions about farming issues to receive possible solutions from agricultural experts, and “Agri-eyes”, a farm environment monitoring system which can support farmers’ decision–making: both proved to be successful in helping the farmers.

Almost half of studies on mobile phones and tablets focused on African countries and their use of SMS, voice messages or WhatsApp for agro-food sales or health advice [68,70,72,74]. Chang et al. [68] assessed the existing ICT contribution in connecting farmers to the market in Senegal: mobile phones were the most widely available technology, but they were still not majorly used as a marketing tool by producers. In Europe, Bacarella et al. [34] focused on mobile phones and tablets as a tool to read QR codes and enhance consumer knowledge on food origin and breeding and cultivation techniques.

In the studies on social media, the most mentioned platform was Facebook, used at different stages of the food supply chain. While urban farmers in Uganda adopted it to exchange agricultural information and to sell their products [71], the EATCambridge Festival used it to facilitate customer–producer, customer-festival and producer-festival interactions [42]. Researchers in the USA used it to improve healthy eating among children [84], while millennials in South Africa posted about their meals helping local restaurants business with e-Word of Mouth [73].

Some of the articles in the literature studied several types of digital technologies: for example, Hearn et al. [77] included 21 of them. The ICT tools covered in their study belonged to the three categories of “App”, “Website/Platform” and “Social Media” and they were compared to describe emerging innovations in urban food systems according to their technical, discursive and social components. The digital technologies were aimed at increasing local and sustainable food consumption, but in particular they focused on providing information on food origin, foraging, food waste reduction, and cooking as well as reinforcing networks of growers and eaters.

### 3.5. Aims of Digital Technologies

The digital technologies analysed in the literature have one main objective: selling and buying agro-food products (42% of studies) (Table 5). This category included articles studying how digital technologies helped trade between producers and consumers.

The second most popular aim of digital technologies (32%) is networking with consumers, a concept linked with the sense of belonging that marketing strategies create among consumers. For online marketing, the most effective strategies were not solely focused on food promotion, but on fostering the networking action of users sharing food information and reviews of food places [32,40,42,45,47,69,73,77].

The third most popular digital technology aim (30%) is health advice. The focus on healthy consumption appeared consistently stronger among Apps. About a third of these studies focused on Apps explored the role of technology in nutritional strategies [38,49,55,76,80,81,83,85].

The fourth most popular aim of digital technologies is food delivery, a topic addressed by 28% of articles. Despite requiring a consistent logistics effort, delivery is increasingly adopted by various types of food retailers, as several platforms and Apps analysed in the literature help small-scale producers to share the organisation and costs of logistics [32,40,41,46,51,78,79]. Food delivery options are increasingly adopted within the HORECA sector; Thamaraiselvan et al. [66] studied food delivery Apps and their perception among Indian consumers with the aim of identifying the drivers for the acceptability of future food delivery platforms.

Providing information on food is the fifth most popular aim and it encompassed various digital technologies presenting the characteristics of food being produced, sold and delivered. Information on food seasonality, sustainability and prices were included in separate categories.

Networking among farmers is the sixth most popular aim. It included both studies on how common social media tools (Facebook and WhatsApp) are used among farmers to exchange information [71], but also articles on lesser-known solutions such as “Hello Tractor”, a software that allows farmers to rent each other′s equipment [29].

The seventh aim, more consistently present among Apps, was restaurants mapping. Hearn et al. [77] analysed the App “Eat St.” which provides an interactive map where users can find food trucks in many cities in the US and Canada. The website “Eat Well Guide” includes an interactive map (“Eat Well Everywhere”) with the localisation of stores, restaurants, bakers, Community Supported Agriculture (CSA) and butchers selling local produce in the US [77].

The remaining digital technologies aims were: helping with farm management, providing travel information, networking with the local community and among all chain actors, providing information on local farms, on agricultural produce prices, on food sustainability, on seasonal food, and organising local farm visits and food waste management.

### 3.6. Section of the Agro-Food Chain where Digital Technologies Operate

Most of the analysed studies focused on consumption (51%) (Table 6). The vast majority consisted of articles on healthy eating, therefore, this category was divided into two sub-categories: “Healthy consumption”, which included research on how digital technologies can help improving citizens′ healthy food choices, and “Food consumption”, which included studies on consumers′ behaviours and eating patterns influenced by digital technologies. The former sub-category consists of the studies on digital technologies used in the nutritional field to stimulate healthy consumption among specific populations or segments of the population. Ball et al. [76] studied an App supporting healthy food habits among socioeconomically disadvantaged women in Australia while Murthy et al. [61] used voice messages to reach the same goal in India. Indigenous communities were the target of two studies aimed at creating culturally appropriate nutrition interventions in Australia [80] and in Canada [85]. Pieniak et al. [38] analysed how to increase healthy food consumption among young adults in Poland, while Bakırcı-Taylor et al. [84] focused on fruits and vegetables consumption among young children in the USA. The “Food consumption” sub-category includes articles that addressed the perceptions towards the use of ICT tools of members of a veg box scheme in the UK [32] and of Food Assembly hubs in Italy [45,47] through interviews, surveys and focus groups. In both cases, consumers were subject to offline interactions with producers and other veg box scheme members, as well as online interactions in the form of blogs, newsletter and producers′ stories [32,45,47].

Besides consumption, production and distribution are the two sections of the agro-food chain where most digital technologies operate, with 37% and 32% of the studies respectively. In the “Production” category fell articles about digital technologies supporting collaborative networks among producers, sharing agricultural information to improve production and helping farmers cooperate for logistics. Studies in the “Distribution” category addressed the role of ICT tools in increasing sustainable consumption by making local food accessible to consumers, for instance through online maps and social media presence.

The third most popular sector is “Retail”, different from “Distribution” as it only includes retail companies and not producers’ selling directly or groups of consumers’ buying in bulk. As mentioned in 3.4, Wang et al. [67] analysed consumers behaviours on four major online shopping platforms in China: Taobao, Tmall, Jingdong and #1Store. Srividya [52] focused on Kirana stores in India, aiming at describing the socio-demographic characteristics of online shoppers.

When considering the whole body of literature, the articles on the role of digital technologies in the HORECA sector were only 12%, but this share reaches 20% when taking into account solely the studies on Apps [59,62,63,66,77]. Oborin [63] studied how the adoption of new technologies could increase the restaurant management efficiency and provide better service quality to customers. For example, local automation systems were recommended for their potential to manage more accurately both the order of service and the supply of ingredients in the kitchen [63]. The digital technology studied by Nikmawati et al. [62] is an offline educational App delivering content about traditional foods of the region of Cireundeu in Indonesia. Albeit being a food information App, it was listed in the HORECA category as it is specifically targeted to students of culinary arts, which makes a difference both in how the information is delivered and in how the students will work.

Tourism was the least researched category. Half of the articles in this field (three out of six) were App-related [48,53,54]. Yen and Zaaba [53] developed an Android App called “Malaycious” whose main function was to map local restaurants in Malaysia. Their App consisted of six main modules: augmented reality for food location, a photographic food diary made by users, a Smart Budget option to select restaurants based on price, a list of top ten Malaysian foods to try, the promotion of Malaysia Food Festival and geographical filtering. The App developed by the study of Fauzi et al. [54] was also focused on restaurant location but in a specific region of Malaysia, Sarawak. More recently, Lee et al. [48] also developed an App prototype called “Local Foodie” using mobile augmented reality (MAR) to encourage local food consumption among tourists in Finland.

**Figure 4:**
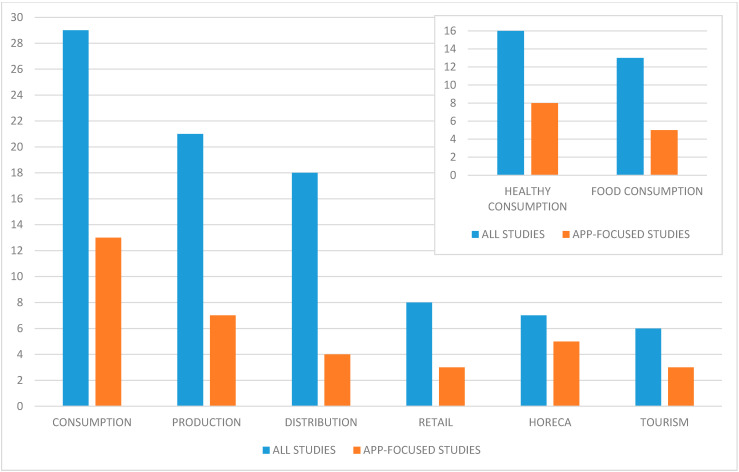
Section of the agro-food chain (*n* of studies). Note: research studies that focused on more than one section were counted more than once.

The sections of the agro-food chain proportionally using more Apps as technology were consumption (especially healthy consumption), HORECA and tourism (Figure 4). Apps are therefore more present in those sectors nearer to the final consumer, being easy and quick to use.

### 3.7. Digital Technologies’ Key Features

**Figure 5:**
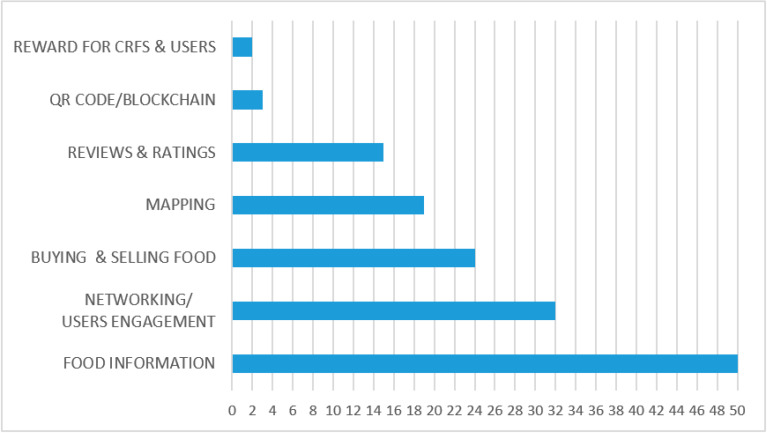
Digital technologies key features (*n* of studies). Note: research studies that focused on more than one feature were counted more than once.

From the literature review it emerged that the key feature of digital technologies was the easy access to information they granted. As shown in Figure 5, “Food information” was the most common feature of digital technologies (88% of the studies). This finding supports that all stakeholders in the food supply chain have an interest in sharing or receiving information. On one hand, thanks to websites, Apps and other ICT tools, agro-food producers, restaurants and nutritionists could share content with customers (or potential ones) in a fast and low-cost manner [42,44,71,73,82]. On the other hand, digital technologies allowed interested customers to easily access food information and share their knowledge with other people, building the sellers′ reputation through electronic word of mouth. Apps granted an even more immediate access to information as supported by Joosse and Hracs [35] who described a Swedish App which allowed consumers to scan a product with their smartphone to know if it was produced in Sweden or not.

Some studies also reported that the use of digital technologies can positively impact profits of agro-food companies [40,41,45,46,47,51,52,54,56,57,60,63,64,65,66,67,69,79]. Digital technologies granted an increase in profits by expanding the customer and consumer base as well as by allowing a higher bargain power to farmers in setting the price (due to the elimination of middlemen). Some authors, however, argued that establishing a pricing model for online transactions which allows good profit margins for producers while being appealing for consumers has proven difficult [39,78]. Results support that the success of business models such as that of the Food Assembly laid in the mix between knowledge sharing on their website and on-site interactions, especially in terms of customers′ purchase frequency [47]. Similarly, Kurnia et al. [78,79] argue that ICT technologies like the Open Food Network can support sustainable practices among food hubs, and that regional supply chains could benefit from the higher coordination allowed by the platform. The most significant challenges are products′ distribution, and difficulty in cooperation among local farmers. Della Gala and Reed [40] found that in the case studies analysed (Stroudco Food Hub and the Dean Forest Food Hub, in the South of England) selling through the Open Food Network meant more efficiency for the producers, and easy access to affordable local food for consumers.

Networking and user interaction were also found to be playing an important part, being discussed in 56% of studies. Networking was guaranteed across the food supply chain: in the study about the Food Assembly in Italy, De Bernardi et al. [45] claimed that “socially and environmentally driven community-oriented platforms can benefit from online interactions” as they facilitate the development of trust towards the network. Kurnia et al. [78] seconded this finding by arguing that “community engagement and empowerment” were benefits often cited by producers using the Open Food Network platform in Australia. The App developed by Henriques and Kock [69] enabled smallholders to interact with traders, retailers, consumers, and each other thus encouraging the emergence of sustainable and fair regional markets.

The “Mapping” feature was present in 33% of articles and it showed a consistent growth in recent years since 2017. Maps are a default in e-commerce platforms for the stores or producers’ locations [29,40,45,47,67,77,78,79]. However, their recent spread is linked to social media, where their use as location tags is a crucial marketing tool to reach a wider client base [64,73].

Blockchain and QR codes were a minor feature and mainly linked to Apps, but of growing interest in the latest years. Hearn et al. [77] described the “Harvest Mark” App, which provided a food traceability system allowing consumers to scan a QR code with their smartphones and receive information about the participating fruit, vegetable and poultry brands, including where the food was grown and what kinds of seeds were used. Syaglova [65] described the case of a Russian food company using blockchain technology to certify the quality of the food they sell.

Other features for successful digital technologies which increase customers′ satisfaction and engagement were security of online payments; punctuality and quality of food delivery; and the use of videos and photos to promote businesses.

While describing digital technologies, some authors also addressed the barriers related to their use [35,39,46,60]. Hossain et al. [60] mentioned farmers′ perception of high technological barriers when trying to sell their products in online buying groups. Ievoli et al. [46] added that the lack of broadband in rural areas was a challenge for many. Another issue peculiar of the agro-food sector is the perishability of agro-food products, which demands quick and accurate supply chain operations, made more complex online [39]. Optimising logistics operations in a cost-effective way is crucial for the success of short supply chains. Effective logistics should involve a number of stakeholders [39]. On the consumer side, the challenge is trust: not being able to see the products first-hand, consumers are unsure of what they are buying, and remain critical of the usefulness of Apps [35].

## 4. Conclusions

The present article aimed at analysing the current literature on digital technologies for purchasing and consumption in urban and regional agro-food systems. The systematic review fulfilled this aim by analysing both the studies on ICT tools and the analysed tools themselves, classifying them according to their key characteristics.

The body of articles on this topic has been steadily growing in the last decade and will probably continue to expand, making further research increasingly relevant. The IT field has proven to be a leading sector in this area of study, taking up the challenge of addressing current issues such as local consumption and healthy eating. The articles’ predominant focus on urban areas supported the idea that digital technologies could strengthen the urban-regional links, as claimed by international agro-food institutions.

Apps, especially those aimed at selling and buying agro-food products, emerged as the most popular digital technology in this research. Consumption, production and distribution were the sections of the agro-food chain where most of these digital technologies operate, providing consumers with information on agro-food products.

The present review supported that the opportunities created by digitalisation are manifold, as stated by the FAO [1]. Digital technologies reinforce the connection between urban and regional food systems by eliminating the middlemen and allowing for a direct contact between producers and consumers, which is usually more difficult to achieve in urban contexts. Moreover, access to Apps, online platforms and websites enhanced the linkages among agro-food chain stakeholders: while the FAO [1] focused on international connectivity, the present review found that the strongest links emerge at a local level. However, none of the articles in the literature addressed how authorities can check the quality and the safety of food sold directly to consumers, making it an interesting issue for further studies. The creation of an online community has proven crucial in enhancing networking among consumers and between them and farmers, radically changing the food shopping experience from both sides. While not being substitute for in person exchanges, online interactions help create a sense of community through sharing food choices and food shopping experiences. Digital technologies, through mapping and reviews, have made it easier for consumers to find local producers and restaurants. They also help maintain these new habits by providing further information and a service that is easy to use and fits with consumers′ busy lifestyles. Mobile phone Apps are particularly fitting for this as they are able to target consumers and farmers, helping the former with sustainable consumption and the latter with farm marketing.

In this review, digital technologies were proven crucial in addressing the issues of asymmetry of information along the food supply chain and of consumers′ detachment towards the food they eat. These are the focuses of the recently published European Green Deal, which aims at using digitalisation as a tool for improving communications of products characteristics to consumers [5]. How to do this at best, could be the topic of further research.

As digital technologies do not bring positive or negative values per se, they must be considered as tools providing resonance and importance to the content they carry. Their transformative potential is in the hands of the users: the issue of access to digital technologies, as pointed out by FAO [1], needs to be addressed. Unequal access to digital technologies could cause long-term disparities hindering the economic development of rural areas worldwide. Thus, the FAO Digital Council committed to a considerable effort in an even access to basic ICT tools for rural communities [1].

Further research is also needed to quantify digital technologies′ impact on citizens′ and consumers′ approach towards urban and regional agro-food systems′ sustainability. Citizens′, consumers′ and food producers’ actual use of digital tools has to be delved into in order to increase ICT tools efficacy and to enhance their transformative power of the agro-food systems.

The present study has some limitations. Firstly, only articles in English were included, excluding studies in other languages. Future research may provide further findings on digital technologies developed in other languages. Secondly, articles were selected from two databases, while the use of other sources is recommended. In particular, repository of latest conference proceedings, dissertations, research and committee reports, government reports, may improve a systematic review’s comprehensiveness. Finally, only articles published until April 2020 were included. Given the speed of evolution in the IT field, further updates may be recommended to timely capture the role of the fast-evolving digital technologies’ phenomenon in the agro-food system.

## Figures and Tables

**Table 1:** Description of the search string and respective database.

Database	Search String	*n* of Studies Identified
Scopus	TITLE-ABS (mobile OR device OR digital OR “smartphone Application” OR App OR ict AND food OR agro-food OR agri-food AND urban OR local OR regional AND consumer OR consumption OR marketing OR commercialization OR distribution OR selling OR delivery OR trade AND NOT medic*) AND (LIMIT-TO (PUBYEAR, 2020) OR LIMIT-TO (PUBYEAR, 2019) OR LIMIT-TO (PUBYEAR, 2018) OR LIMIT-TO (PUBYEAR, 2017) OR LIMIT-TO (PUBYEAR, 2016) OR LIMIT-TO (PUBYEAR, 2015) OR LIMIT-TO (PUBYEAR, 2014) OR LIMIT-TO (PUBYEAR, 2013) OR LIMIT-TO (PUBYEAR, 2012) OR LIMIT-TO (PUBYEAR, 2011) OR LIMIT-TO (PUBYEAR, 2010))	370
Web of Science	TOPIC: (mobile OR device OR digital OR “smartphone Application” OR App OR ict AND food OR agro-food OR agri-food) AND TOPIC: (urban OR local OR regional) AND TOPIC: (consumer OR consumption OR marketing OR commercialization OR distribution OR selling OR delivery OR trade) NOT TOPIC: (medic*) TIMESPAN: 2010–2020	398

**Table 2:** Geographical distribution of the research studies.

	Continent	*n* of Studies	References
1	Europe	21	[29,30,31,32,33,34,35,36,37,38,39,40,41,42,43,44,45,46,47,48,49]
2	Asia	21	[29,36,37,50,51,52,53,54,55,56,57,58,59,60,61,62,63,64,65,66,67]
3	Africa	12	[29,32,36,37,68,69,70,71,72,73,74,75]
4	Oceania	9	[29,36,37,76,77,78,79,80,81]
5	America	8	[29,36,37,77,82,83,84,85]

Research studies that focused on more than one country were counted more than once.

**Table 4:** Types of digital technologies.

	Type of Digital Technology	*n* of Studies	References
1	Apps	25 (44%)	[29,35,38,48,49,51,53,54,55,56,57,59,62,63,65,66,69,75,76,77,80,81,83,85]
2	Websites and platforms	20 (35%)	[29,32,33,39,40,41,44,45,46,47,50,51,52,60,67,69,77,78,79,84]
3	Mobile phones and tablets	9 (16%)	[30,34,58,61,68,70,72,74,84]
4	Social media	6 (15%)	[42,64,71,73,77,84]
5	Print and broadcast media	2 (4%)	[68,82]
6	Videogame	1 (2%)	[43]
7	Modern kitchen appliance	1 (2%)	[31]

Research studies that focused on more than one type of digital technology were counted more than once.

**Table 5:** Aims of digital technologies.

	Aim of Digital Technology	*n* of Studies	References
1	Selling and buying agro-food products	24 (42%)	[29,32,35,39,40,41,45,46,47,50,51,52,56,57,59,60,63,65,66,67,71,77,78,79]
2	Networking with consumers	18 (32%)	[32,34,36,40,42,45,46,47,50,64,68,69,71,73,77,78,79,82]
3	Health advice	17 (30%)	[30,37,38,43,49,55,58,61,62,72,74,76,80,81,83,84,85]
4	Food delivery	16 (28%)	[32,39,40,41,46,51,52,56,59,63,65,66,67,77,78,79]
5	Food information	13 (23%)	[32,34,35,36,38,42,55,62,64,68,76,77,82]
6	Networking among farmers	9 (16%)	[29,45,46,47,50,51,60,69,71]
7	Restaurant mapping	8 (14%)	[48,53,54,59,64,66,73,77]
8	Farm management	8 (14%)	[29,50,51,56,60,69,70,75]
9	Travel information	7 (12%)	[44,46,48,53,54,64,77]
10	Networking with the local community	6 (11%)	[40,42,45,47,78,79]
11	Networking among all chain actors	5 (9%)	[29,41,51,57,69]
12	Local farms information	4 (7%)	[35,45,46,47]
13	Information on agricultural produce prices	3 (5%)	[33,50,69]
14	Sustainability information	2 (4%)	[31,35]
15	Seasonal food information	1 (2%)	[85]
16	Local farm visit	1 (2%)	[46]
17	Food waste management	1 (2%)	[77]

Research studies that focused on more than one aim were counted more than once.

**Table 6:** Section of the agro-food chain.

	Section of the Agro-Food Chain	*n* of Studies	References
1	Consumption	29 (51%)	[29,30,31,32,35,36,37,38,40,41,43,45,47,49,55,58,59,61,72,74,76,77,78,79,80,81,83,84,85]
1a	Healthy Consumption	16	[30,37,38,43,49,55,58,61,72,74,76,80,81,83,84,85]
1b	Food Consumption	13	[29,31,32,35,36,40,41,45,47,59,77,78,79]
2	Production	21 (37%)	[29,31,32,33,40,41,42,45,46,47,50,51,56,57,60,69,70,71,75,77,82]
2	Distribution	18 (32%)	[29,31,32,39,40,41,46,47,50,51,56,60,68,71,77,78,79,82]
3	Retail	8 (14%)	[29,31,33,39,52,57,65,67]
4	HORECA	7 (12%)	[59,62,63,64,66,72,77]
5	Tourism	6 (10%)	[42,44,46,48,53,54]

Research studies that focused on more than one section were counted more than once.

## Data Availability

Not applicable.
